# A Novel Assay for Phosphoserine Phosphatase Exploiting Serine Acetyltransferase as the Coupling Enzyme

**DOI:** 10.3390/life11060485

**Published:** 2021-05-26

**Authors:** Francesco Marchesani, Erika Zangelmi, Stefano Bruno, Stefano Bettati, Alessio Peracchi, Barbara Campanini

**Affiliations:** 1 Department of Food and Drug, University of Parma, 43124 Parma, Italy; francesco.marchesani@unipr.it (F.M.); stefano.bruno@unipr.it (S.B.); 2 Department of Chemistry, Life Sciences and Environmental Sustainability, University of Parma, 43124 Parma, Italy; erika.zangelmi@unipr.it; 3 Department of Medicine and Surgery, University of Parma, 43125 Parma, Italy; stefano.bettati@unipr.it; 4 Institute of Biophysics, National Research Council, 56124 Pisa, Italy

**Keywords:** phosphoserine phosphatase, serine detection, enzymatic assay, serine phosphorylated pathway, serine acetyltransferase, Ellman’s reagent, 5,5’-dithionitrobenzoate (DTNB), malachite green assay

## Abstract

Phosphoserine phosphatase (PSP) catalyzes the final step of de novo L-serine biosynthesis—the hydrolysis of phosphoserine to serine and inorganic phosphate—in humans, bacteria, and plants. In published works, the reaction is typically monitored through the discontinuous malachite green phosphate assay or, more rarely, through a continuous assay that couples phosphate release to the phosphorolysis of a chromogenic nucleoside by the enzyme purine nucleoside phosphorylase (PNP). These assays suffer from numerous drawbacks, and both rely on the detection of phosphate. We describe a new continuous assay that monitors the release of serine by exploiting bacterial serine acetyltransferase (SAT) as a reporter enzyme. SAT acetylates serine, consuming acetyl-CoA and releasing CoA-SH. CoA-SH spontaneously reacts with Ellman’s reagent to produce a chromophore that absorbs light at 412 nm. The catalytic parameters estimated through the SAT-coupled assay are fully consistent with those obtained with the published methods, but the new assay exhibits several advantages. Particularly, it depletes L-serine, thus allowing more prolonged linearity in the kinetics. Moreover, as the SAT-coupled assay does not rely on phosphate detection, it can be used to investigate the inhibitory effect of phosphate on PSP.

## 1. Introduction

Phosphoserine phosphatase (PSP; EC 3.1.3.3) is a dimeric enzyme that catalyzes the final and irreversible step of the phosphorylated pathway of L-serine biosynthesis, which contributes most of endogenous L-serine in humans [1]. In the brain, the role of L-serine biosynthesis is crucial for two key reasons. Firstly, L-serine is poorly transported across the blood–brain barrier, so that the brain must primarily rely on local L-serine production for protein biosynthesis [2]. Secondly, L-serine is a precursor of sphingomyelin and of the neuromodulators D-serine and glycine [3], both required for brain development and function. Glycine is produced from L-serine by serine hydroxymethyltransferase [4], whereas D-serine is produced by serine racemase [5], a pyridoxal 5-phosphate-dependent enzyme strongly regulated by effectors such as ATP [6,7], divalent metals [8], and S-nitrosylation [9,10]. 

In consideration of the importance of PSP in the brain, it is not surprising that inborn deficiencies of PSP, as well as of the other enzymes in the phosphorylated pathway, are associated with neurological symptoms [11,12,13,14,15,16]. In mice, the knockout of both copies of the PSP-encoding gene is apparently lethal [17], further confirming the crucial biological role of this enzyme and of the phosphorylated pathway.

**Figure 1:**
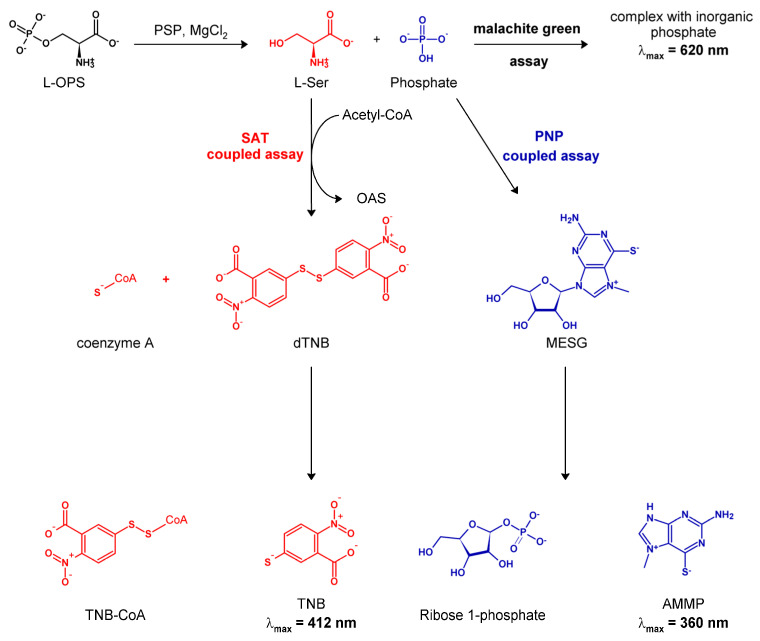
Scheme of the mechanism of the three different enzymatic assays used for monitoring the PSP reaction, as discussed in the text.

Our group is currently involved in a thorough analysis of the functional properties of human PSP, which requires the extensive use of kinetic techniques to measure its activity. To this end, we first tried to identify the most efficient and versatile assay(s) to monitor the PSP reaction. The majority of kinetic studies conducted on PSP (from whatever source) are based on the detection of the released inorganic phosphate through the discontinuous malachite green assay [18,19,20,21] (Figure 1). This assay, first introduced in the 1960s by Itaya and Ui [22] and further developed over time, has become a standard method in the study of phosphatases. Nevertheless, the assay remains tedious, practically inapplicable to monitor fast reactions, and often prone to the precipitation of the dye, which adds to the overall margin of error in determining kinetic parameters. The molybdenum blue assay, which is also occasionally used [23,24], suffers from similar problems. Furthermore, the malachite green reagent is notoriously toxic [25]. 

A more recent continuous assay, originally developed by Webb [26], detects phosphate through a coupling enzyme, purine nucleoside phosphorylase (PNP; EC 2.4.2.1). PNP employs the phosphate ion to break the glycosidic bond in a chromogenic nucleoside (Figure 1). This assay has since been used in a number of studies on phosphatases (e.g., [27,28,29]), including one on bacterial PSP [30], but failed to become dominant in the field.

Furthermore, in the context of our experimental goals, it was highly desirable to complement the assays that monitor the release of phosphate with methods to detect the second product of the phosphatase reaction, L-serine. In fact, PSP is known to be inhibited by its product L-serine with a complex mechanism [19,31]. Therefore, a coupled assay with an enzyme that consumes the inhibitory product L-serine would be best suited for the determination of the intrinsic kinetic parameters of PSP. Moreover, a continuous assay based on the detection of L-serine could overcome the typical issues of discontinuous assays, such as the malachite green assay, in monitoring fast enzyme reactions.

Accordingly, we envisaged that a suitable continuous assay could couple the PSP reaction to that of bacterial serine acetyltransferase (SAT; EC 2.3.1.30). SAT catalyzes a committed step in the cysteine biosynthetic pathway in bacteria and plants, the activation of L-serine by *O*-acetylation to yield *O*-acetylserine (OAS) [32,33], which in turn is the substrate of the last enzyme of the pathway, *O*-acetylserine sulfhydrylase [34]. The co-product of the SAT reaction, CoA-SH, rapidly and stoichiometrically reacts with 5,5′-dithiobis (2-nitrobenzoic acid) (DTNB, Ellman’s reagent) to yield its colored dianion 2-nitro-5-thiobenzoate (TNB^2−^), which can be quantified through absorption at 412 nm [35,36,37] (Figure 1).

In this work, we describe the SAT-coupled assay, and we compare in detail its performances with those of the malachite green and PNP-coupled assays. While the results obtainable with the three assays were grossly comparable, the SAT-coupled assay proved particularly reliable and lacked most of the drawbacks of the other two methods. Some examples of the advantages of this assay (such as the possibility of using it to investigate the inhibitory effect of phosphate on PSP activity) will be presented. Other applications of the assay will be also discussed.

## 2. Materials and Methods

### 2.1. Reagents

Acetyl-CoA was purchased from AppliChem (Darmstadt, Germany). 7-Methyl-6-thioguanosine (MESG) was purchased from Cayman (Ann Arbor, MI, USA). The malachite green reagent, PNP, and all other chemicals were purchased from Merck (Darmstadt, Germany).

### 2.2. Protein Expression and Purification 

Human PSP, cloned in the NdeI/BamHI sites of a pET28a vector, was expressed in *E. coli* BL21(DE3) cells (Novagen^®^, Merck, Darmstadt, Germany). This clone was a kind gift of Drs. Emile Van Schaftingen and Maria Veiga-da-Cuhna (De Duve Institute, UCLouvain, Bruxelles) [38]. For protein expression, the bacterial culture was grown at 37 °C in 1 L of LB medium supplemented with kanamycin (50 µg/mL) until 0.7–0.8 OD_600_, at which point the temperature was reduced and the induction was started with 0.2 mM isopropyl-β-D-1-thiogalactopyranoside (IPTG) at 20 °C o/n. Cells were then collected by centrifugation (7200× *g* for 10 min at 4 °C) and stored at −20 °C. For protein purification, the cell pellet was resuspended in a lysis buffer (25 mM HEPES, 100 mM NaCl, 5 mM TCEP, 1 mg/mL lysozyme, 0.2 mM benzamidine, 0.2 mM PMSF, and 1.5 µM pepstatine pH 7.4), sonicated and centrifuged (18,000× *g* for 30 min at 4 °C). The N-terminal His_6_-tagged PSP was purified by IMAC on a Talon Superflow^TM^ resin (Cytiva, Marlborough, MA, USA). The fractions containing PSP were pooled and extensively dialyzed against 25 mM HEPES, 100 mM NaCl, pH 7.4 to remove imidazole. The protein was then concentrated, frozen in liquid nitrogen, and stored at −80 °C. The protein yield was 23 mg per liter of culture.

SAT from *Haemophilus influenzae* (HiSAT), cloned in the NdeI/BamHI sites of a pET28a vector, was expressed in *Escherichia coli* Tuner^TM^ (DE3) cells (Novagen^®^, Merck, Darmstadt, Germany) and purified by IMAC as described elsewhere [39]. The hexahistidine tag was removed with thrombin, and the protein was further purified by IMAC. The protein was stored in aliquots at −80 °C in 50 mM Tris, 50 mM NaCl, 1% glycerol, pH 7.5. The concentrated stock solution (24 mg/mL) was stable at 4 °C and could be frozen and thawed multiple times, while the diluted solutions (0.5 mg/mL) were prepared fresh and discarded after use. 

The biosynthetic serine/threonine deaminase from *E. coli* (product of the *ilvA* gene) was expressed and purified as described in [40].

### 2.3. Activity Assays

All the assays were carried out in buffer H (50 mM HEPES, 100 mM KCl, 3 mM MgCl_2_, pH 7) at 37 ± 0.4 °C. Additional reagents were added as required by the specific assays, as detailed below. 

#### 2.3.1. Malachite Green Discontinuous Assay 

The malachite green discontinuous activity assay was performed in buffer H with the addition of BSA to 0.1 mg/mL. L-OPS (L-*O*-phosphoserine) was used at concentrations ranging from 22 to 600 µM, depending on the experiments. The buffered solution was pre-incubated at 37 ± 0.4 °C, and the reactions were triggered by the addition of PSP. The mixtures were sampled over time, and the reactions were stopped by the addition of 100 µL of the malachite green reagent. As indicated by the manufacturer, thermal or chemical inactivation of the enzyme was not required, as the malachite green reagent stops the enzyme reactions. Samples were incubated with the malachite green reagent for 30 minutes at room temperature in the dark. The absorbance of the molecular complex between inorganic phosphate and the malachite green reagent was measured using a HALO LED 96 microplate reader (Dynamica, Livingstone, UK) set at 620 nm. The assay is linear up to 2000 pmoles of phosphate, i.e., 100 μM phosphate in the assay mixture. 

#### 2.3.2. PNP-Coupled Continuous Assay 

To perform the PNP-coupled assay, we used a commercially available bacterial PNP. The protein cake was dissolved in buffer T (containing 20 mM Tris, 100 mM NaCl, 10% glycerol, 1 mM TCEP, pH 8). The mixture was then centrifuged, and the supernatant dialyzed against buffer T. Dialysis is required to remove inorganic phosphate, which interferes with the assay. 

The concentration of PNP was determined based on its absorbance at 278 nm using the extinction coefficient of 8940 M^−1^·cm^−1^ assessed by ProtParam (https://web.expasy.org/protparam/). The MESG solutions were prepared in 100% DMSO under nitrogen atmosphere, as suggested by the manufacturer. The concentration of MESG was determined by the extinction coefficient at 331 nm of 32,000 M^−1^·cm^−1^ at pH 7 [41]. The specific activity of PNP was evaluated at the same temperature and in the same solution used for the PSP assay, with the additional presence of 0.7 mM potassium phosphate. A unit of PNP is defined as the amount of enzyme that converts 1 µmole of MESG in 2-amino-6-mercapto-7-methylpurine (AMMP) in one minute in the presence of 0.7 mM potassium phosphate at 37 °C, pH 7.

The PNP-coupled continuous assays were performed in buffer H supplemented with MESG to 100 µM and PNP to 300 mU. L-OPS was used at different concentrations ranging from 10 to 500 µM, depending on the experiments. The concentration of MESG and the enzymatic units of PNP were modified in some experiments, as specified. Each reaction was set up in a microcuvette (l = 1 cm) and pre-incubated for 3 minutes at 37 °C in the spectrophotometer (Cary 4000, Agilent, Santa Clara, CA, USA) cuvette holder, reading the baseline absorbance at 360 nm. After 3 minutes of incubation, the reactions were triggered by the addition of PSP, and the initial velocity was estimated within the stationary phase by following the absorbance at 360 nm. The velocity was then converted to the rate of AMMP production using a Δε of 11,200 M^−1^·cm^−1^ at 360 nm between MESG and AMMP [41]. The detection range of the assay is 5–90 μM phosphate. 

#### 2.3.3. Serine/Threonine Deaminase-Coupled Continuous Assay

The serine/threonine deaminase-coupled continuous assay (Figure S1) was performed in buffer H with different added concentrations of coupling enzyme, ranging from 3 to 11 µM, L-OPS to 300 µM, NADH to 100 µM, and 60 U/mL of L-lactate dehydrogenase (one unit will reduce 1 μmole of pyruvate to L-lactate per minute at pH 7.5 at 37 °C). Since serine/threonine deaminase is activated by its product ammonia, 2 to 30 mM concentrations of NH_4_Cl were used in order to activate the enzyme [40]. 

Each reaction was set up in a microcuvette (l = 1 cm) pre-incubated in the spectrophotometer cuvette holder for 3 minutes at 37 °C before reading the baseline absorbance at 340 nm. After 3 minutes of incubation, the reactions were triggered by the addition of PSP or L-OPS, and the initial velocity was estimated from the linear phase of the kinetic trace. The variation in absorbance at 340 nm was used to calculate the rate of the reaction using the extinction coefficient of NADH of 6220 M^−1^·cm^−1^.

#### 2.3.4. SAT-Coupled Continuous Assay 

The SAT-coupled continuous assays were performed in buffer H with the addition of acetyl-CoA to 170 µM, DTNB to 0.5 mM, and 430 mU of HiSAT. L-OPS was used at different concentrations ranging from 10 to 500 µM, depending on the experiment. The units of HiSAT were modified in some experiments, as specified. Each reaction was set up in a microcuvette (l = 1 cm) pre-incubated in the spectrophotometer cuvette holder for 3 minutes at 37 °C before reading the baseline absorbance at 412 nm. After 3 minutes of incubation, the reactions were triggered by the addition of PSP, and the initial velocity was estimated from the linear phase of the kinetic trace. The variation in absorbance at 412 nm was used to calculate the rate of product synthesis using the extinction coefficient reported for TNB at pH 7 (14,100 M^−1^·cm^−1^) [35,36,37]. The specific activity of HiSAT was estimated at the same temperature and in buffer H, with the additional presence of 170 µM acetyl-CoA, 0.5 mM DTNB, and 1 mM L-serine [42,43]. A unit of HiSAT is defined as the amount of enzyme able to convert 1 µmole of acetyl-CoA in free coenzyme A in one minute in the presence of 1 mM L-Ser to give OAS at 37 °C, pH 7. The detection range of the assay is 4–70 μM phosphate.

## 3. Results

The current work was motivated by the quest for a convenient, continuous assay to monitor the PSP reaction based on the detection of L-serine to complement existing detection methods based on the release of inorganic phosphate. We first attempted to exploit the coupled reactions of L-serine deaminase (that would convert L-serine into pyruvate) and lactate dehydrogenase, as described in Materials and Methods (see also Figure S1). However, the development of this assay was abandoned because the bacterial deaminase is relatively inefficient, even in the presence of high concentrations of ammonia, a positive effector. Moreover, the addition of serine/threonine deaminase in high amounts (up to 11 µM) to the reaction mixture under the conditions of our assays resulted in protein precipitation (data not shown). 

Therefore, we developed a new assay that exploits SAT as the coupling enzyme in the presence of DTNB. The rationale of the assay is schematically shown in Figure 1 and the pertinent technical details are provided in the Materials and Methods section. To validate this assay, as well as to evaluate its range of applicability, advantages, and potential shortcomings, we set up a systematic comparison of its performances with those of the two assays employed in previous kinetic studies (malachite green and PNP-coupled assay).

**Figure 2:**
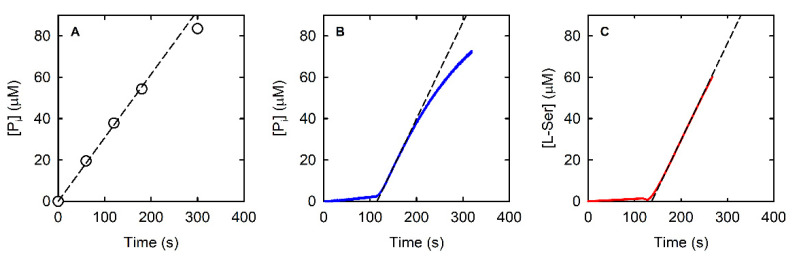
Examples of PSP reactions kinetics in the presence of 0.3 mM L-OPS using three different enzyme assays, the malachite green discontinuous assay (**A**—9.3 nM PSP), the PNP-coupled continuous assay (**B**—15.7 nM PSP in the presence of 0.1 mM MESG and 300 mU of PNP), and the SAT-coupled continuous assay (**C**—12.5 nM PSP in the presence of 0.17 mM acetyl-CoA and 430 mU of HiSAT). Circles (Panel A) and continuous lines (Panels B and C) represent experimental data, whereas dotted lines represent the fitting to a linear equation used to calculate initial rates.

**Figure 3:**
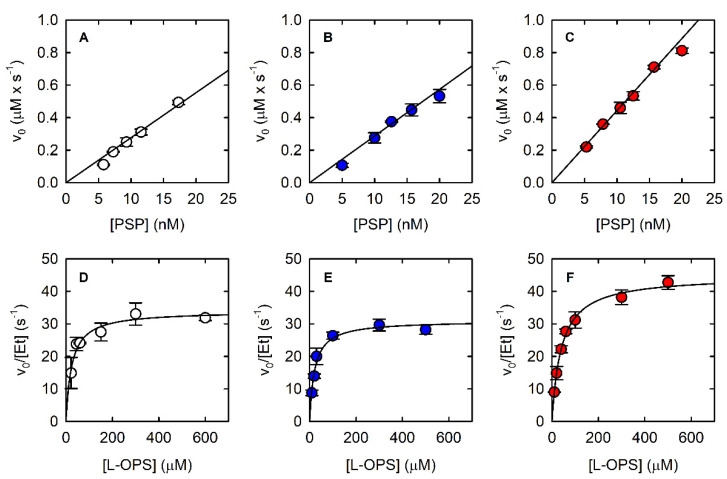
Dependence of the initial velocity of the PSP-catalyzed reaction on PSP concentration at saturating concentration of L-OPS (0.5–0.6 mM) using the malachite green assay (**A**), the PNP-coupled assay (**B**), and the SAT-coupled assay (**C**). Dependence of the initial velocity of PSP-catalyzed reaction on L-OPS concentration using the malachite green assay (**D**—9.3 nM PSP), the PNP-coupled assay (**E**—15.7 nM PSP), and the SAT-coupled assay (**F**—12.5 nM PSP). PNP-coupled assays were carried out in the presence of 3.6 µM PNP, which corresponds to 320 mU. SAT-coupled assays were carried out in the presence of 800 nM SAT, which corresponds to 430 mU. The solid lines through the data points in Panels D–F are nonlinear least-squares fits of the data to the Michaelis–Menten equation. The parameters derived from such fittings are reported in Table 1.

The three assays were compared under an uniform set of conditions (pH 7, 37 °C). The results are summarized in Figure 2 and Figure 3, as well as in Table 1. In the following sections, the features of each assay are analyzed in detail. 

### 3.1. The Classic Discontinuous Assay: Malachite Green Assay

The amount of phosphate released in the reaction catalyzed by 9.3 nM PSP in the presence of 0.3 mM L-OPS (i.e., a saturating concentration, vide infra) was measured through the malachite green assay (Figure 2A). The malachite green assay was linear up to a phosphate concentration of about 60 μM, i.e., up to a consumption of about 20% of the initial L-OPS (Figure 2A).

The fitting of the linear part of the kinetics allowed the calculation of a slope of 0.3 μM/s, and despite the known limits of discontinuous assays for measuring accurate catalytic parameters, we could determine the *K*_M_ and the *k*_cat_ by fitting the dependence of the initial velocity on L-OPS concentration by the Michaelis–Menten equation (Figure 3D, Table 1); calculated parameters are in good agreement with published data [21]. The observed reaction rate depended linearly on the concentration of PSP up to about 20 nM (Figure 3A).

### 3.2. A Continuous Assay of Phosphate Release (after Some Troubleshooting): The PNP-Coupled Assay

To set up a continuous assay for the determination of the catalytic parameters of the PSP-catalyzed reaction, we decided to optimize the method proposed by Webb in 1992 [26,44] using commercial PNP and the MESG chromogenic substrate. PNP dissolved in buffer T shows an absorption maximum at 260 nm, possibly due to the presence of nucleic acids in the commercial PNP preparation, thus preventing the determination of the correct protein concentration (Figure S2A). To obviate this problem, the concentration of PNP was calculated by densitometry on SDS-PAGE (data not shown). The dependence of the reaction rate on PSP concentration was measured using 0.5 μM PNP in a 20–80 nM PSP range (Figure S2B). The observed rate increased linearly up to 40 nM PSP while linearity was lost at higher concentrations. However, unexpectedly, the rate did not increase with further addition of PNP, an indication that the rate was not limited by the amount of coupling enzyme. Moreover, the linear part of the dependence had a slope significantly lower than the one measured using the malachite green assay, an indication that the PNP-coupled assay was underestimating the actual reaction rate. We further observed that upon addition of 0.5 μM PNP to the reaction mixture, about 40% of MESG was consumed, suggesting that the PNP preparation contained phosphate and MESG consumption was leading to substrate shortage, hence limiting the overall reaction rate. Indeed, in the original paper [26], a MESG concentration of 200 μM was recommended. However, the *K*_M_ of PNP for MESG is 70 μM and, thus, 200 μM is not completely saturating, and the measured rate is likely to decrease fast upon initial MESG consumption. Furthermore, because of the high extinction coefficient of the chromogenic substrate at 360 nm (11,200 M^−1^·cm^−1^) the use of such an elevated concentration is not recommended, due to high absorbance values (>2 OD), which exceed the linearity limit of most spectrophotometers. 

**Figure 4:**
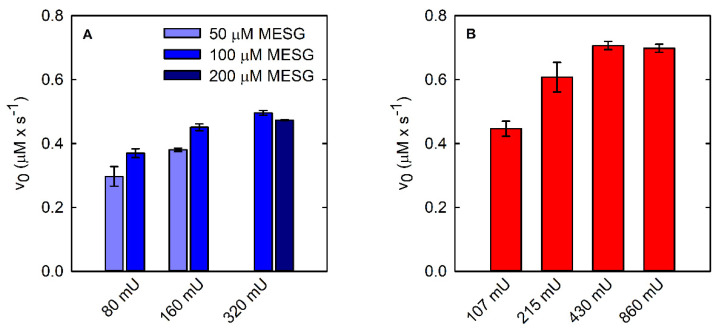
Coupling of PSP reactions (15.7 nM final concentration) using two different continuous enzymatic assays: PNP-coupled assay (**A**) and SAT-coupled assay (**B**).

We dialyzed the PNP stock solution against buffer T and recorded the absorption spectrum, which was correctly centered at 278 nm (Figure S2A). When the dialyzed preparation was used to measure the reaction rate as a function of PSP concentration, we obtained a linear dependence up to 20 nM PSP, with a threefold increase in slope in comparison to the previous experiment. In Figure 2B, the kinetic trace collected at 0.3 mM L-OPS in the presence of 15.7 nM PSP, 3.6 µM (300 mU) PNP, and 0.1 mM MESG is reported. At variance with the malachite green assay, the trace is linear up to about 30 μM concentration, an indication that the amount of MESG, rather than that of L-OPS, limits the duration of the steady-state regime. However, the initial slope of the kinetic trace is 0.47 µM/s, in good agreement with the one measured with the malachite green assay, once normalized for PSP concentration. We deemed it important to assess the effect of both PNP and MESG concentration on the correct reaction coupling, and we thus measured the rate of the PSP-catalyzed reaction at different combinations of PNP and MESG concentration (Figure 4A). 

Indeed, we observed that the initial velocity of phosphate production by PSP depends on both PNP and MESG concentrations at low PNP units (Figure 4A). Briefly, 320 mU PNP with either 100 or 200 μM MESG does not limit the reaction rate and was thus used in the following experiments. Under the optimized conditions, it was possible to measure the dependence of the reaction rate on PSP concentration (Figure 3B) and the catalytic parameters (Figure 3E, Table 1). The parameters are in very good agreement with those calculated using the malachite green assay. 

### 3.3. Advantages and Applications of the SAT-Coupled Assay

The SAT-coupled assay detects L-Ser instead of phosphate, with two main advantages: (i) it removes L-Ser, a known inhibitor of PSP [38], and (ii) it allows the determination of the effect of phosphate on the catalytic activity. In this assay, L-Ser is acetylated by SAT using acetyl-CoA, and the produced CoA-SH spontaneously and instantaneously reacts with DTNB to give a mixed CoA-TNB disulfide and TNB. The latter absorbs at 412 nm with an extinction coefficient of 14,100 M^−1^·cm^−1^. The higher extinction coefficient of TNB compared to that of AMMP (14,100 vs. 11,200 M^−1^·cm^−1^) makes the SAT-coupled assay slightly more sensitive than the PNP-coupled assay. Furthermore, the extinction coefficient of TNB depends less on pH, for pH values greater than 7 (Figure S3). Indeed, the extinction coefficient of AMMP at 360 nm drops from 11,200 M^−1^·cm^−1^ at pH 7 to 9300 M^−1^·cm^−1^ at pH 7.6, further decreasing the sensitivity of the assay, while the ε of DTNB slightly increases under the same conditions. The PSP reaction can be effectively coupled to the SAT reaction, as demonstrated by the kinetic trace that is comparable to those obtained by the malachite green and the PNP-coupled assays (Figure 2C). The slope is slightly higher than that measured with the malachite green and the PNP-coupled assays, likely because L-Ser consumption alleviates the effect of product inhibition on PSP activity. SAT does not limit the overall reaction rate when 430 mU or more is used (Figure 4B). 

**Figure 5:**
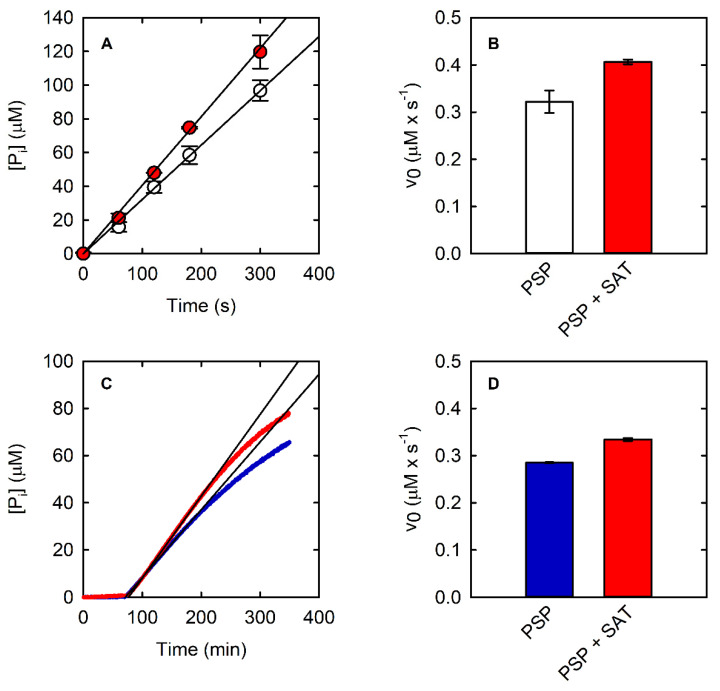
(**A**) Kinetics of PSP (10 nM) at saturating concentration (0.6 mM) of L-OPS obtained with the malachite green assay in the absence (open circles) and presence (red circles) of SAT (430 mU). Black lines are the fitting of data points within the linear part of the kinetic using a linear regression. (**B**) Initial velocity of PSP in the same conditions as (**A**), in the absence and presence of SAT (430 mU). (**C**) Kinetics of PSP (10 nM) at saturating concentration of L-OPS (0.5 mM) obtained with the PNP-coupled assay in the absence (blue line) and presence (red line) of SAT (430 mU). The black lines are the fitting of data points within the linear part of the kinetic using a linear regression. (**D**) Initial velocity of PSP in the same conditions as (**C**), in the absence and presence of SAT.

The dependence of the initial rate on PSP concentration is linear within a range comparable to that of the other assays (Figure 3C), and the kinetic parameters calculated from the fitting of the Michaelis–Menten plot are also comparable (Figure 3F, Table 1). However, both *K*_M_ and *k*_cat_ are larger than those calculated with the two assays that detect phosphate instead of L-Ser. To assess whether the removal of L-Ser was responsible for this effect, the malachite green assay and the PNP-coupled assay were carried out in the presence and absence of 800 nM SAT and of 170 µM Acetyl-CoA. Acetyl-CoA was added in both conditions to single out the effect of SAT on produced L-serine (Figure 5). Acetyl-CoA alone did not have any effect on PSP activity (data not shown). Indeed, the initial rate of the PSP reaction is 25% and 18% greater in the presence of SAT than in its absence for the malachite green assay and the PNP-coupled assay, respectively. The less pronounced effect of SAT on the PNP-coupled assay might reflect the greater complexity of the reaction mixture, which may bring about some marginal secondary effects. These findings agree with the larger *k*_cat_ obtained with the SAT-coupled assay in comparison to the values obtained with the other two enzyme assays, confirming that SAT removes the product inhibition by L-serine.

**Figure 6:**
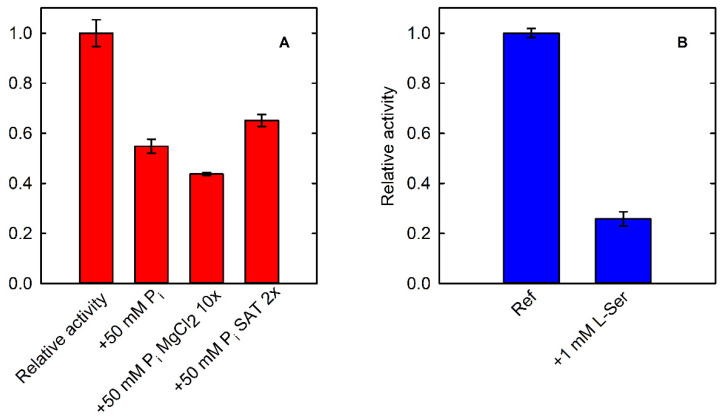
(**A**) Fractional activity of PSP (15.7 nM, in the presence of 40 µM L-OPS) in the absence (Ref) and presence of 50 mM phosphate evaluated by using the SAT-coupled assay (430 mU SAT). The effect of phosphate was also assessed in the presence of a 10-fold molar excess of MgCl_2_ in comparison to normal assay conditions, to avoid a possible chelating effect on the divalent metal (+50 mM P_i_ MgCl_2_ 10x). The assay in the presence of 50 mM phosphate was also performed by doubling the SAT concentration (+50 mM P_i_ SAT2x), to confirm that the inhibition was related to PSP and not to the coupling enzyme. (**B**) Fractional activity of PSP (15.7 nM–20 µM L-OPS) in the absence (Ref) and presence of 1 mM L-Ser evaluated by using the PNP-coupled assay (300 mU PNP).

Another relevant feature of the SAT-coupled assay is the possibility of testing the effect of inorganic phosphate on the activity of PSP, which is not possible when using phosphate-based assays. As a matter of fact, PSP is 50% inhibited by 50 mM phosphate (Figure 6A). The inhibitory effect is not associated to the chelation of Mg^2+^ by phosphate ions, as demonstrated by an experiment carried out in the presence of a 10-fold higher concentration of MgCl_2_ in comparison to standard assay conditions. SAT is poorly inhibited by 50 mM phosphate (Figure S4). Therefore, we also checked the effect of phosphate on PSP by doubling the concentration of the coupling enzyme, resulting in negligible effect and confirming that the observed inhibition is related to PSP.

We also demonstrated that the application of both phosphate-based and L-serine-based assays affords complementary information on PSP activity. Indeed, it is possible to investigate the effect of L-serine by using the assay that detects phosphate (Figure 6B) and also to characterize the effect of phosphate using the assay based on the detection of L-serine (Figure 6A). 

## 4. Conclusions

We have devised and validated a new continuous assay to monitor the activity of PSP by exploiting SAT as a coupling enzyme. To properly evaluate the pros and cons of this method, we systematically compared its performances with those of two other assays employed in previous studies. The catalytic parameters estimated with the new assay are consistent with those obtained with the malachite green and the PNP-coupled assay, while eliminating most of their drawbacks. Among these drawbacks, we would like to signal the need to check for the presence of phosphate and other contaminants in commercial PNP preparations and, in case they are present, to remove them. Most importantly, L-serine consumption by SAT relieves PSP from feedback inhibition, granting prolonged linear phases, minimizing artifacts in enzyme kinetics, and allowing the calculation of the correct *k*_cat_. Moreover, we also investigated the effects of the products L-serine and phosphate using the PNP-coupled assay and the SAT-coupled assay, respectively, showing that both products regulate the activity of PSP. Interestingly, SAT selectively uses the L-enantiomer of serine, and thus the assay will not be affected by the presence of D-serine. The coupled assay developed here is also easily adaptable to high-throughput screening conditions, in analogy with [42], and can thus be very useful for the identification and characterization of inhibitors [31]. In this respect, the main advantage of the SAT-coupled assay over the malachite green assay is the continuous operation mode, and, over the PNP-coupled assay, is that the detection wavelength in the visible region is less likely to suffer from interferences, especially when using standard, plastic 96-well plates. 

## Figures and Tables

**Table 1:** Kinetic parameters of PSP-catalyzed dephosphorylation of L-OPS obtained using different assays. The parameters are reported as +/- standard error of the regression.

	Malachite Green Assay	PNP-Coupled Assay	SAT-Coupled Assay
***K*_M_ (** **μM)**	24.4 ± 4.4	21.1 ± 3.3	40.3 ± 2.8
***k*_cat_ (s^−1^)**	33.9 ± 1.3	31.0 ± 1.2	44.8 ± 0.9
***k*_cat_/*K*_M_ (M^−1^** **s^−1^)**	(1.39 ± 0.30) × 10^6^	(1.47 ± 0.29) × 10^6^	(1.11 ± 0.10) × 10^6^

## Supplementary Materials

The following are available online at https://www.mdpi.com/article/10.3390/life11060485/s1, Figure S1: Scheme of the mechanism of the serine/threonine deaminase (TD)-coupled continuous assay. Figure S2: Effect of PNP dialysis on its applicability in the coupled assay; Figure S3: Effect of pH on MESG/AMMP and DTNB/TNB; Figure S4: Effect of phosphate in SAT activity.
