# Supplementary material for: A Novel Assay for Phosphoserine Phosphatase Exploiting Serine Acetyltransferase as the Coupling Enzyme
Source: Life (Basel). 2021 May 26;11(6):485. doi: 10.3390/life11060485 (PMC8229081; doi:10.3390/life11060485)
Supplement: Supplementary file 1 [file life-11-00485-s001.zip › life-1222498-supplementary.pdf]

Supplementary material of A Novel Assay for Phosphoserine Phosphatase Exploiting Serine Acetyltransferase as the Coupling Enzyme

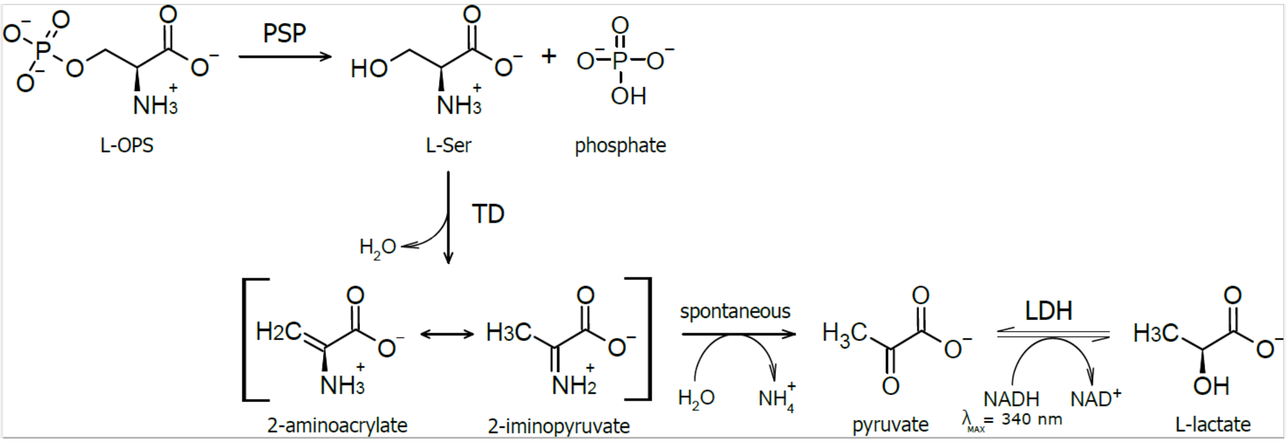

Figure S1. Scheme of the mechanism of the serine/threonine deaminase (TD)-coupled continuous assay.

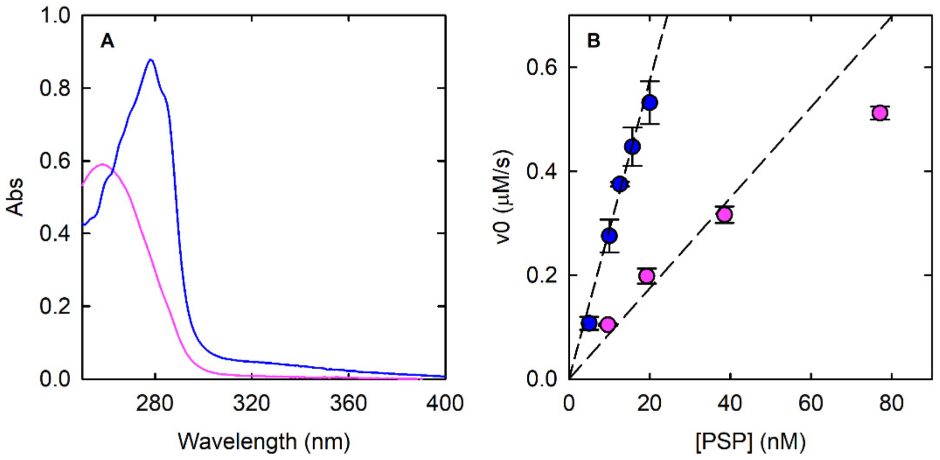

Figure S2. (**A**) Absorption spectra of PNP before (purple) and after (blue) dialysis. (**B**) Dependence of the initial velocity of the phosphatase reaction on PSP concentration in the presence of saturating concentrations of L-OPS (0.3-0.5 mM). PNP was dissolved in buffer T and used either before (purple dots) or after (blue dots) extensive dialysis against the same buffer. The concentration of PNP used was 0.5 μM (purple dots) and 3 μM (blue dots). As mentioned in the results section, the use of higher concentrations of undyalized PNP does not increase the rate of PSP reaction.

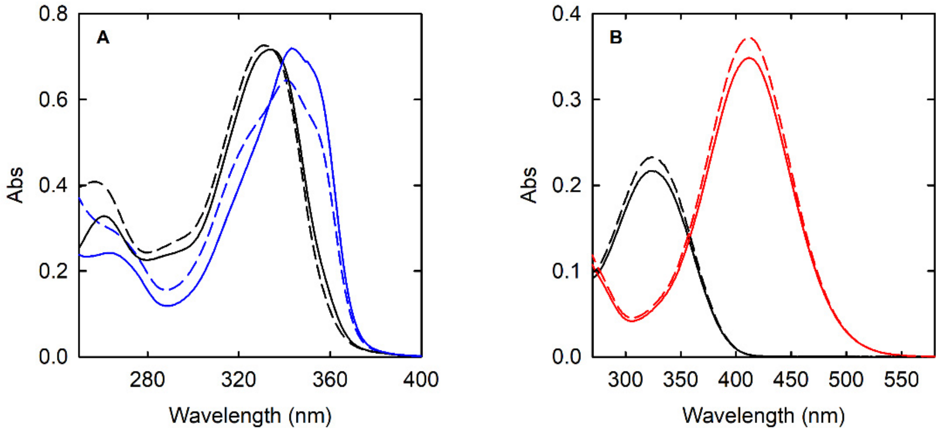

Figure S3. (**A**) Absorption spectra of MESG before (black) and after (blue) the addition of PNP (end point) at pH 7.0 (continuous lines) and pH 7.6 (dashed lines). (**B**) Absorption spectra of DTNB before (black) and after (red) the addition of 1 mM DTT at pH 7.0 (continuous lines) and pH 7.6 (dashed lines).

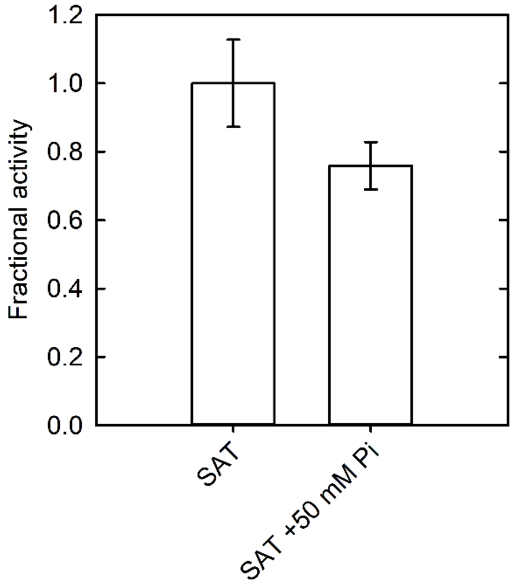

Figure S4. Effect of 50 mM phosphate in buffer H on SAT activity (4 nM SAT) in the presence of 1 mM L-Ser.
